# Study on Spraying Construction Method of a Non-Water Reacting Polymer Layer in the Tunnel

**DOI:** 10.3390/ma15124138

**Published:** 2022-06-10

**Authors:** Bo Sun, Chengchao Guo, Yu Chen, Xuanxuan Chu, Xue Ma

**Affiliations:** 1 School of Civil Engineering, Sun Yat-sen University, Guangzhou 510275, China; sunb27@mail2.sysu.edu.cn (B.S.); guochch25@mail.sysu.edu.cn (C.G.); chenyu68@mail.sysu.edu.cn (Y.C.); 2 Southern Marine Science and Engineering Guangdong Laboratory, Zhuhai 519082, China; 3 Nottingham Transportation Engineering Centre, Faculty of Engineering, University of Nottingham, Nottingham NG7 2RD, UK; 4 School of Civil Engineering, Southwest Jiaotong University, Chengdu 610031, China; xuema@my.swjtu.edu.cn

**Keywords:** tunnel, polymer, damping layer, spraying construction

## Abstract

Non-water reacting double-component foamed polyurethane has been increasingly used in the field of transportation. Particularly, it is recognized that a polymer damping layer between tunnel linings and surrounding rocks can improve the seismic performance of tunnels. To facilitate the application of this polymer in tunnels, a spraying construction method of polymer damping layers was proposed. The polymer damping layer was prepared with hydraulic spraying equipment, and the construction process included the pre-treatment of the tunnel base surface, the pressure control of the spraying equipment, the adjustment of the spray gun working parameters and spraying quality control. In this paper, the effects of material ratio, material temperature, environmental factors (i.e., temperature, humidity and wind speed), spraying pressure and spray gun parameters (i.e., speed, distance from the sprayed surface and spray angle) on the layer formation were investigated. Thus, spraying parameters for better spraying performance were recommended. This study will provide technical support for polymer damping layer construction in the seismic design of tunnels.

## 1. Introduction

Recently, with the implementation of China’s western development strategy, an increasing number of tunnel projects are constructed. Consequently, some tunnels are located in high-intensity active fault areas, which belong to strong earthquake-prone areas. Once a severe earthquake happens, serious damage will occur in the tunnel structure with disastrous consequences [1,2]. Therefore, it is necessary to apply earthquake-resistant methods and accordingly develop supporting construction methods.

The construction of a damping layer in the tunnel is an important damping measure. The common damping materials in the existing research include rubber plate [3], foam concrete [4,5], fiber concrete [6], polymer composite [7], etc. However, the above materials have the defects of temperature sensitivity, poor durability and low preparation efficiency, and there are few reports on their construction technology. At present, the non-water reacting polyurethane polymer material developed by our group (hereinafter referred to as polymer material) has the characteristics of safety and environmental protection, rapid response and adjustability, impermeability and waterproof capability and good durability [8,9,10]. Combined with the polymer grouting technology, it has been widely used in the reinforcement and maintenance of tunnels, highways, dams and other infrastructure [11,12]. In addition, the material is a cellular structural material [13] with good flexibility and damping performance [14,15], which is included in the alternative material for tunnel vibration reduction and isolation. Therefore, further research on the construction technology of polymer materials in tunnel engineering will help to solve the difficult problem of tunnel seismic engineering. Based on the previous research, at a smaller density but larger thickness of the material, the damping performance of the material was better. However, according to the specific project, there also exists the most suitable density and thickness of the damping layer [16]. Therefore, this paper focused on studying the construction technology of the polymer damping layer.

Polyurethane spraying is a representative technology in structural engineering. The principle of this technology is similar to that of tunnel shotcrete construction and tunnel shotcrete membrane waterproof construction. However, due to different spraying materials and uses, this technology has high requirements for temperature regulation, spraying pressure and spraying layer quality [17,18,19]. Currently, the polyurethane spraying construction technology has been successfully used in the fields of waterproof and thermal insulation of building structures [20], thermal insulation of cold storage and grain depot [21], thermal insulation and anti-corrosion of pipeline and trenchless repair [22,23]. However, the study on its utilization in large tunnel engineering to construct earthquake-resistant structures has been rarely conducted or is still in a theoretical stage. Non-water reacting polyurethane polymer materials have been used as an alternative material to construct the damping layer for tunnel vibration reduction and isolation, due to their good flexibility, durability and seismic performance. Polymer material-based damping layers through spraying technology will provide an alternative method for the seismic design of tunnels. During the spraying process, the process parameters have an impact on the coating quality, varying from each parameter. It is necessary to analyze their effects on the coating quality and control their variation range.

This study aims to develop a spraying construction method of polymer damping layers for tunnel engineering. The effects of material ratio, material temperature, environmental factors (i.e., temperature, humidity and wind speed), spraying pressure and spray gun operational parameters (i.e., speed, distance from the sprayed surface and spray angle) on the performance of spraying construction technology in constructing the polymer material-based damping layer were studied. Then, the suitable spraying parameters or their ranges can be determined and applied to actual tunnel projects. The spraying construction process of the polymer damping layer was also proposed. The research results can provide technical support for the seismic construction of mountain tunnels in strong earthquake-prone areas.

## 2. Materials and Methods

### 2.1. Physical Model

**Figure 1:**
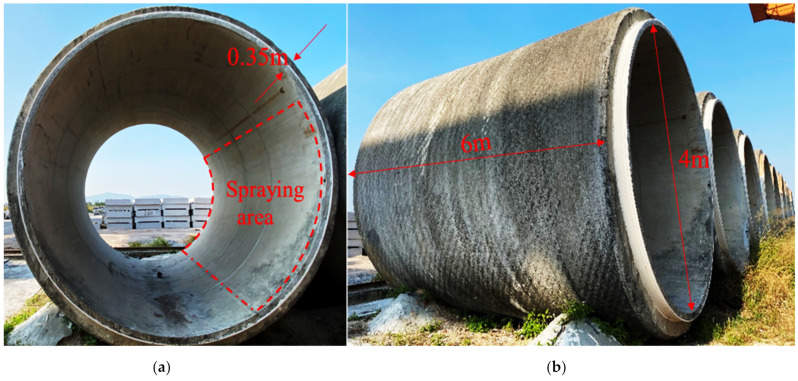
Spraying test model: (**a**) cross section; (**b**) vertical section.

To better reflect in-situ conditions of tunnels, a large diameter concrete pipe was used to simulate the curved surface structure of tunnel linings with a dimension of 4 × 6 × 0.35 m (diameter × length × thickness). The pipe segment is a prestressed steel cylinder concrete pipe for jacking construction, as shown in Figure 1. The test area was also presented in Figure 1. The test scheme implemented in the tunnel model is shown in Table 1.

### 2.2. Spraying System and Work Principle

**Figure 2:**
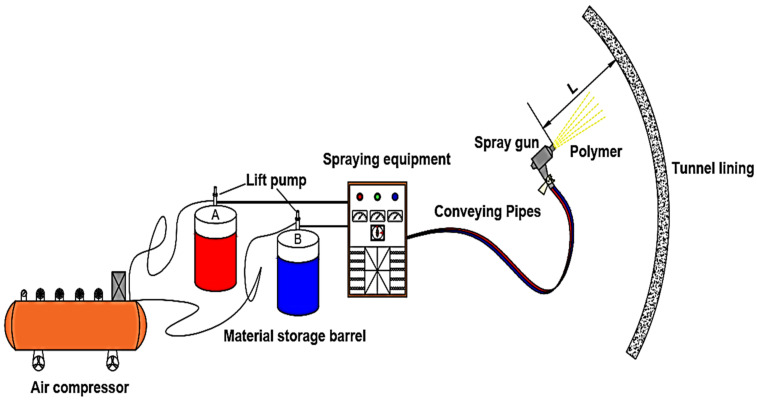
Schematic diagram of polymer spraying construction system and spraying operation.

Generally, the equipment system used for spraying rigid polyurethane foam includes raw material storage and transport system, a metering pump, a heating system, a feeding pipe and a spray gun. The components of the construction equipment and the spraying process are shown in Figure 2.

The portable spraying equipment with hydraulic pressure was used to spray the polyurethane in the test. The basic principle is based on the two lifting pumps of the machine. The air compressor drives the lifting pump to send the raw materials to the host. The host uses the secondary pump (the hydraulic station directly provides power for the secondary pump of the machine) to pressurize the raw materials. Then, it is heated by the heating pot of the main engine and sent to the heating and thermal insulation pipes. Next, the high-pressure mixing is conducted through a spray gun, and the mixture is injected immediately for construction. In addition, it should be equipped with an air compressor system that can meet the requirements of spraying construction.

### 2.3. Test Procedure and Process

**Figure 3:**
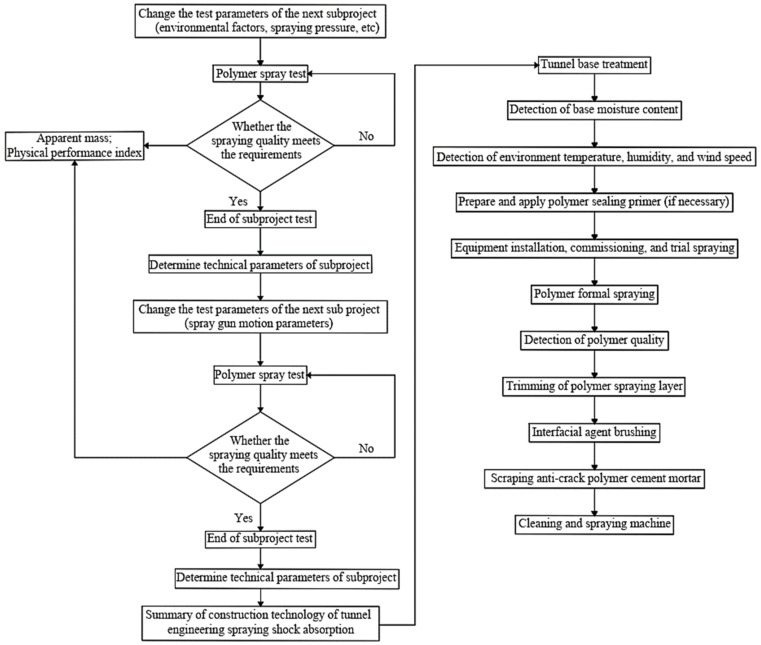
Spraying test process and sequence of each sub-project and construction process of polymer spraying system.

Due to the large testing scale and different requirements for each test, the test method for each sub-project was slightly adjusted. The criterion set for the spraying process is that each polymer damping layer can meet the structural seismic requirements. Figure 3 shows the spraying test process and sequence of each sub-project. The basic test procedure is as follows: (1) set the test parameters of the sub-item (i.e., spraying pressure and initial material temperature); (2) spray the polymer to the predetermined surface; (3) quality evaluation, i.e., evaluate the property variation of the polymer damping layer; (4) according to the termination criteria of the test, the polymer spraying is stopped; (5) finalize the technical parameters and standards of the sub-project. Based on the technical parameters and standards of the previous sub-project, the research on the next sub-project (at varying spray gun motion parameters) was conducted. Thus, based on the results of the overall test, the spraying construction technology of the seismic-adsorption layer for tunnel engineering was proposed.

The whole polymer spraying process was performed following the National standard of China-Technical Code for Rigid Polyurethane Insulation and Waterproof Engineering [24]. Figure 3 shows the construction process of the polymer spraying system. The process mainly includes tunnel base treatment; detection of base moisture content; detection of environment temperature, humidity, and wind speed; preparation and application of polymer sealing primer (if necessary); equipment installation, commissioning, and trial spraying; polymer formal spraying; detection of polymer quality; trimming of polymer spraying layer; interfacial agent brushing; scraping anti-crack polymer cement mortar and cleaning of spraying machine.

## 3. Results and Discussion

### 3.1. Material Ratio

**Figure 4:**
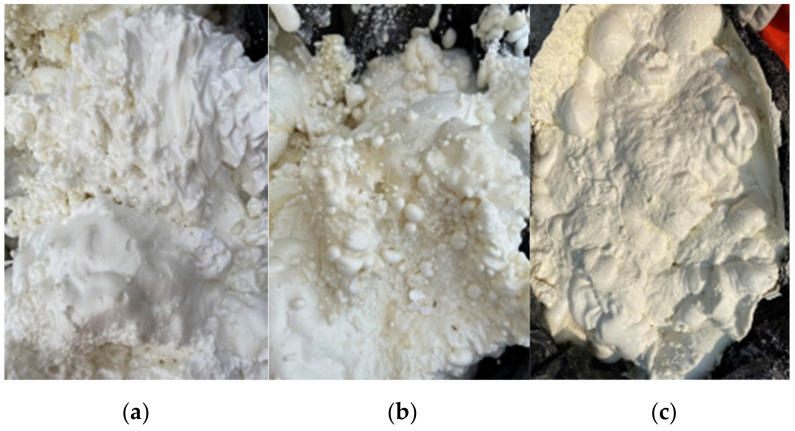
Illustrations of polymer foam at different λ of Components A and B: (**a**) λ > 1, (**b**) λ < 1, (**c**) λ = 1.

Polymer spraying requires that the ratio (λ) of Components A and B (Two components of polymer grout) be 1:1, and the fixed material ratio of the spraying equipment was 1:1. However, due to the large viscosity difference between Components A and B, the λ of 1:1 cannot be guaranteed under the same lifting pressure. When Component A was dominant, the polymer had a low foam density, white color, creamy appearance, low foam strength and soft handle; it was prone to shrink at a low temperature, as shown in Figure 4a. In contrast, when Component B was dominant, the foam exhibited a higher density, darker color and higher strength, and the handle was hard and brittle, as shown in Figure 4b. When λ was 1:1, the foaming showed a normal pattern, as shown in Figure 4c. In these cases, the material ratio should be checked immediately to see whether the filter was blocked and whether the pressure and temperature indicator of the equipment were normal, to ensure the λ accuracy of Components A and B. In these three scenarios, the compressive strength of the polymer with a density of 0.1 g/cm^3^ was 1.6, 2.16 and 2.82 MPa, respectively. However, when Component B was dominant, brittle failure occurred in the material and does not show good flexible energy dissipation performance.

### 3.2. Material Temperature

The material showed lower viscosity at a higher initial temperature. An optimal material temperature can accelerate the chemical reaction speed and also indirectly affect the adhesive strength and the coating quality. In the spraying test, the initial polymer material temperature ($T_{poly}$) was set as 25, 35, 45, 55 and 65 °C by setting the equipment temperature, respectively. The apparent quality and adhesive strength of the coating after spraying were also assessed. Table 2 shows the spraying performance at different initial material temperatures.

The test results show that with the increase in the initial material temperature, the bond strength between the spraying coating and the base first increased at 25–45 °C, and then decreased at 45–65 °C. This is due to the fact that the material reaction is slow at a low material temperature and the foaming is not completely developed inside with slight sagging and splashing, resulting in low fracture strength of the material. On the contrary, the material reaction process is rapid at a higher material temperature with exothermic and adequate heating, which leads to the gasification of moisture in the base layer. Consequently, the tensile bonding strength of the base layer interface was reduced. The debonding time for the coating from the base layer decreased at a higher temperature.

### 3.3. Environmental Factors

(1) Environment and tunnel base temperature

**Figure 5:**
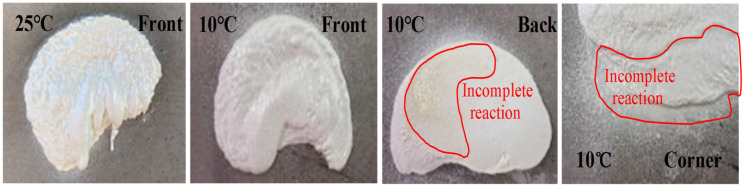
Polymer spraying effects at 25 and 10 °C.

The foaming of the polymer materials was greatly affected by temperature. When the ambient temperature ($T_{en}$) increased, the reaction became faster with less reaction time required for foaming. The foam was homogeneous with almost the same density at the surface and core. When the ambient temperature was low (e.g., below 15 °C), part of the reaction heat can transfer into the surrounding environment. The loss of heat prolongs the curing period of foam, and the shrinkage rate of foam molding increased due to the resulting lower temperature. The amount of derived polymer foam increased. The test results show that the volume of foaming material at an ambient temperature of 10 °C was about 28% smaller than that at 25 °C, as shown in Figure 5. This indicates an increase in the production cost of the foam. It is also found that the spraying base temperature had a great influence on the foaming effect of polymer materials. During the spraying process, when the ambient temperature and the temperature of the tunnel lining base were quite low, the reaction heat was rapidly absorbed by the base after the first spraying of the polymer. Thus, this resulted in incomplete foaming at the bottom and corners and reduced the foaming volume of the material. Therefore, during construction, the process should be reasonably arranged at a higher ambient temperature in order to ensure the foaming rate of polymers.

(2) Humidity of tunnel base

**Figure 6:**
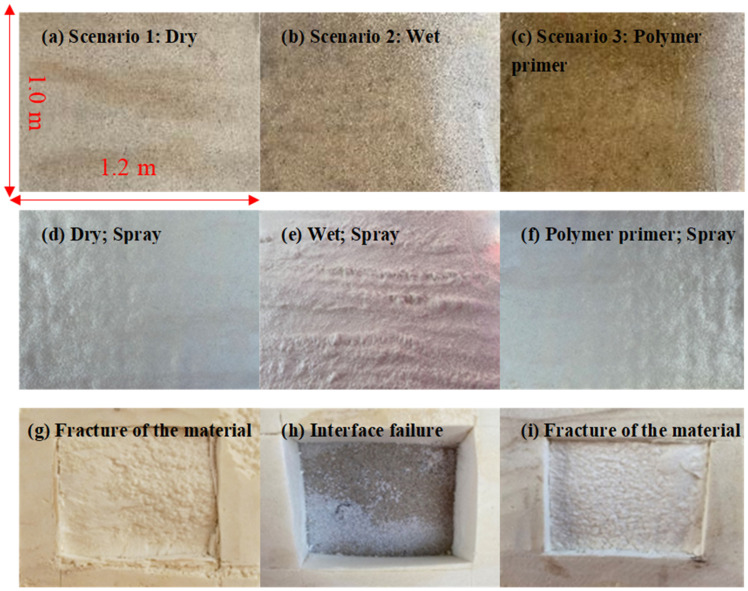
Three scenarios, spraying performance and adhesive strength tests under three scenarios: (**a**–**c**) for Scenarios 1~3; (**d**,**g**) for Scenario 1; (**e**,**h**) for Scenario 2; (**f**,**i**) for Scenario 3.

Polymer foam is a polymer product formed after the two-component mixing reaction of isocyanate and polyol. Among them, the isocyanate component easily reacts with water to form urea. If the urea bond content in the polyurethane increases, the foam will become brittle, and the adhesive stress between the foam and the substrate will decrease. The specification requires that the adhesive strength between foam and base layer be not less than 0.1 MPa. Therefore, the surface of the base layer should be clean and dry, with a relative humidity $\left(w_{base}\right)$ of less than 85% or ideally without water. No construction is allowed on rainy days. The base surface with dew or frost should be removed and dried. The spraying construction of the polymer damping layer and the adhesive strength test of the spraying layer was carried out under three scenarios: Scenario 1, dry base; Scenario 2, wet base; Scenario 3, wet base coated with self-developed polymer primer, as shown in Figure 6. The test results show that a relatively uniform damping layer was formed under Scenarios 1 and 3, while the spraying layer under Scenario 2 was uneven with a large number of bubbles occurring. It can be attributed to the instantaneous gasification of the tunnel base moisture caused by the intensely exothermic material reaction. The adhesive strength test shows that the adhesive strength under Scenarios 1~3 was 401, 2 (failure) and 410 kPa, respectively. The failure position of Scenarios 1 and 3 lay within the material, and thus, the adhesive bond strength between the spraying layer and the base layer was expected to be larger than these measured values. This indicates that the spraying polymer layer had good adhesion performance and can meet the engineering requirements.

(3) Wind speed

**Figure 7:**
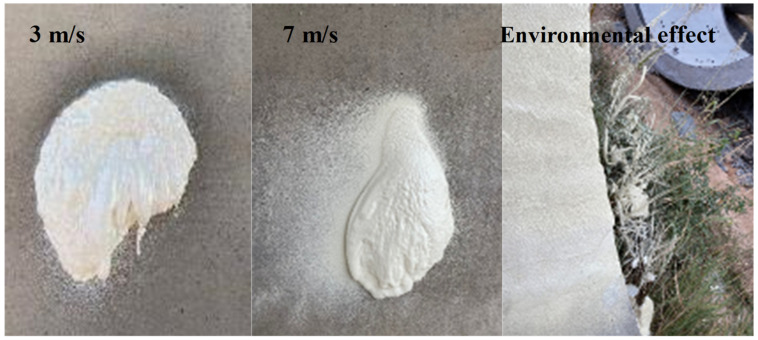
Polymer spraying performance at different $v_{wind}$.

In the process of spraying, the wind speed is required to be below a Level 3 wind, with a speed of 5 m/s. When the wind speed ($v_{wind}$) exceeded Level 3, the heat generated from the reaction rapidly transferred to the surroundings, thus affecting the curing of the polymer foam. The surface of the sprayed layer also became brittle. When $v_{wind}$ was higher, the material particles sprayed in an atomized state were blown away, increasing the loss of raw materials and polluting the environment, as shown in Figure 7. The spray test results show that with the same amount of raw polymer material, the foam volume at a $v_{wind}$ of 7 m/s was about 30% smaller than that at a $v_{wind}$ of 3 m/s. It indicates that the usage of engineering materials will increase for construction at a higher $v_{wind},$ and thus the budget will increase.

### 3.4. Spraying Pressure

**Figure 8:**
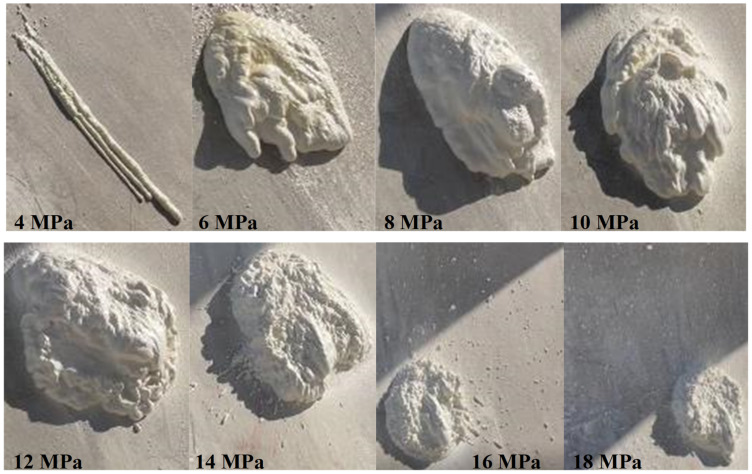
Spraying performance and diffusion radius at different $\sigma_{spray}$.

The spraying results at different spraying pressures ($\sigma_{spray}$) are illustrated in Figure 8 with different diffusion radius and atomization effects summarized in Table 3. The results show that with higher $\sigma_{spray}$, the spraying radius increased gradually, and better atomization effects were achieved. When the pressure was less than 8 MPa, sagging occurred to varying degrees, and it gradually disappeared with the increase in $\sigma_{spray}$. When $\sigma_{spray}$ exceeded 14 MPa, the material splash occurred, which got worse with the increase in $\sigma_{spray}$. Therefore, the suitable $\sigma_{spray}$ range could be 10–12 MPa. The power provided by the hydraulic station in the spraying equipment needs to be adjusted, in order to acquire the appropriate pressurization of the raw material by the secondary pump.

### 3.5. Spray Gun Operational Parameters

The motion parameters of the spray gun mainly include the spray gun speed, spraying distance and spraying angle. Firstly, the reasonable range of each parameter was predetermined according to the spraying effect and experience. Then, tests with different parameter combinations were carried out. Due to the influence of spraying base surface conditions, operator’s experience and other factors, the values of these parameters could be continuously adjusted in real-time according to the spraying effect. Therefore, the main purpose of this test is to determine the reasonable range of the spray gun motion parameters to guide the on-site spraying construction of the tunnel polymer damping layer.

(1) Spray gun speed

**Figure 9:**
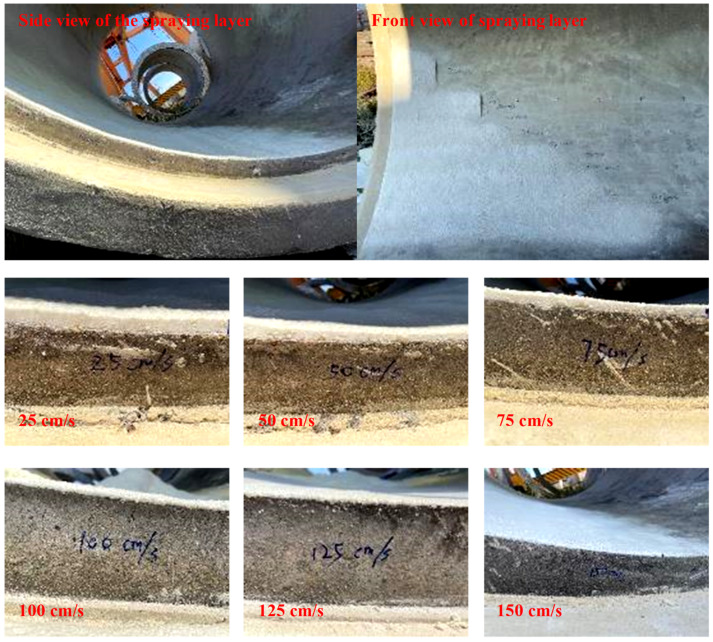
Spraying layer thickness at different $v_{gun}$.

During the spraying test, the speed of the spray gun $(v_{gun}$) needed to be adjusted within the range of 25 to 150 cm/s, and the surface of the coating, the thickness and apparent density of the derived coating after single-layer spraying were observed, as shown in Figure 9 and Table 4. The test results show that as $v_{gun}$ increased, the thickness of the single polymer layer gradually decreased. When $v_{gun}$ reached 150 cm/s, the overall apparent density of the sprayed layer was about 500 kg/m^3^ with uneven patterns.

(2) Spraying distance

**Figure 10:**
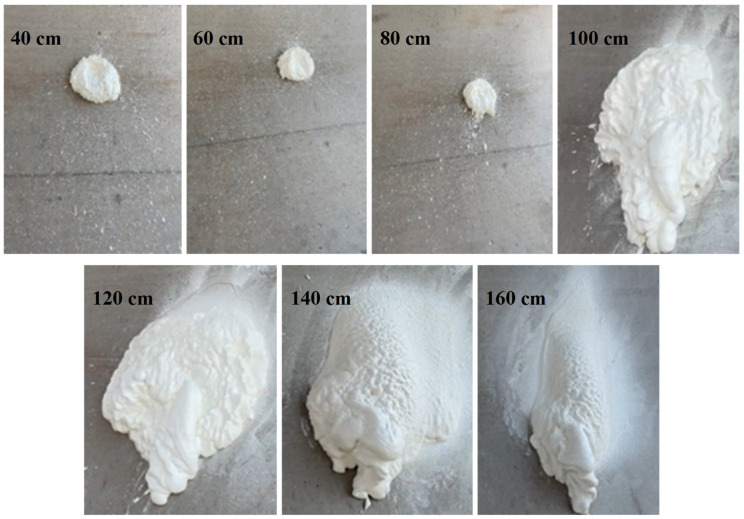
Spraying performance at different $L_{spray}$.

The spraying distance ($L_{spray}$) is the distance between the mixing nozzle of the spray gun and the surface of the sprayed structure. With $L_{spray}$ ranging from 40 to 160 cm, it is observed that the spraying radius increased initially and then decreased, as shown in Figure 10 and Table 5. When $L_{spray}$ was smaller, a serious splash occurred due to the high pressure of the spray gun, resulting in an uneven spraying layer. When $L_{spray}$ was larger, the foggy material was sprayed onto the base surface in a parabola, due to the influence of the self-weight of the material and $v_{wind}$. Thus, the uneven phenomenon of the polymer coating occurred. Therefore, the proper $L_{spray}$ can facilitate obtaining better spraying effects.

(3) Spraying angle

**Figure 11:**
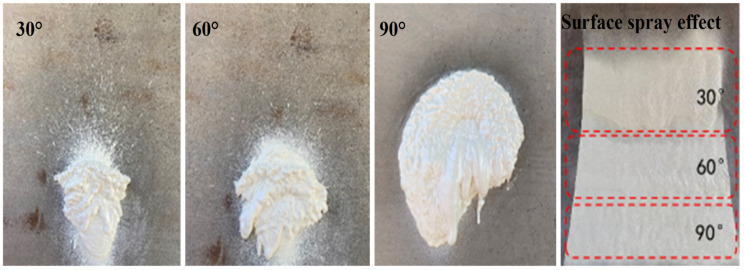
Spraying performance at different $\theta_{spray}$.

In general, when the nozzle was not perpendicular to the base surface, it caused problems, such as uneven spraying surface quality and difficulty in controlling the thickness. The nozzle should be perpendicular to the tunnel base surface (i.e., the spraying angle $\theta_{spray}$ = 90°), as shown in Figure 11. It is allowed that the nozzle is not perpendicular to the base surface, which can be adjusted appropriately according to the actual situation, e.g., the uneven base surface and tunnel auxiliary structures.

### 3.6. Recommended Spraying Parameters for Polymer Damping Layer

**Figure 12:**
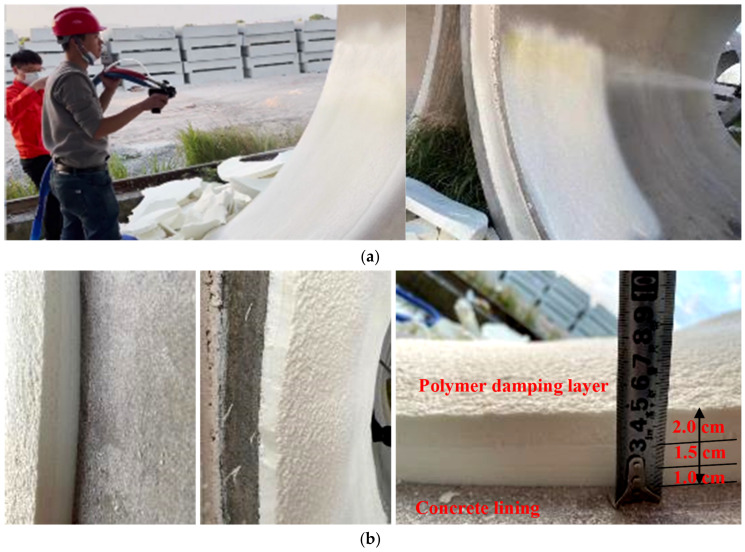
Spraying construction process of polymer damping layer and illustration of layered spraying effects: (**a**) spraying construction process; (**b**) spraying effects.

The test results indicate that an optimal range for the above spraying parameters is possible, in order to achieve the best spraying quality of the polymer damping layer. Based on the above test, the suitable parameter range of the spraying process (Table 6) was proposed to provide a reference for field tests and in-situ construction. The spraying process of the polymer damping layer on the tunnel model and the spraying effect are shown in Figure 12. The spraying construction is conducted in layers: (1) spray the polymer slurry onto the target surface to develop the first layer (i.e., a priming spray), with an advisable thickness of about 1 cm; (2) continue to spray onto the first layer to develop the second layer immediately with a thickness lower than 20 mm; (3) repeat step (2) until the average value of the total thickness reaches the design thickness of the polymer damping layer. Therefore, spraying layers with different thicknesses can be realized by controlling the speed of the spray gun.

## 4. Case Study

### 4.1. Project Overview

The Chongli Tunnel, which is to be constructed, is located in a mountainous area on the north side of Huangtuzui Village, Xiwanzi Town, Chongli District, Zhangjiakou City, Hebei Province, about 500 m to the east of Dajiadaogou Village, Xiwanzi Town, Chongli District, China. The tunnel starts at DK62 + 310.0 and ends at DK67 + 800.0, with a total length of 5490 m and a maximum burial depth of 383.3 m. The tunnel has three inclined shafts, including the No. 1 inclined shaft connected to DK63 + 700, with a total length of 820 m; the No. 2 inclined shaft connected to DK64 + 900, with a total length of 440 m; and the No.3 inclined shaft connected to DK66 + 230, with a total length of 260 m.

The basic peak acceleration of ground vibration in this area is 0.10 g and seismic intensity is Degree VII. Considering the insulation and waterproof performance, two parameters (a polymer spraying thickness of 5 cm and a material density of 0.1 g/cm^3^) were adopted.

### 4.2. In-Situ Application and Results

**Figure 13:**
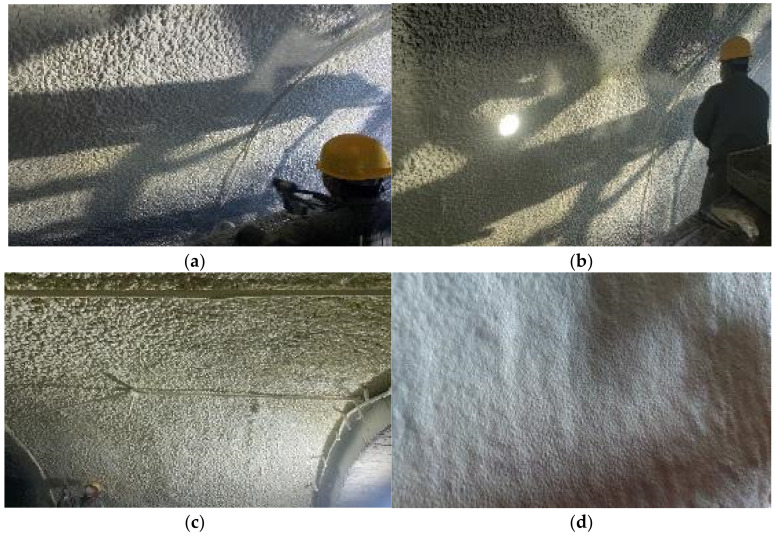
On-site spraying process of polymer materials and spraying effect at different positions: (**a**) spraying process of the first layer; (**b**) spraying process of the second layer; (**c**) spraying effect at the vault; (**d**) spraying effect at the arch waist.

Following the spray procedure in Section 2.3, the polymer spray was applied to the project to be constructed. The spraying process and effects at the vault and arch waist are presented in Figure 13.

In order to quantitatively assess the spraying effects, the characteristics of the material before and after the spray were investigated. The results are presented in Table 7. The results show that the spraying performance is satisfactory to achieve a polymer damping layer with good quality.

## 5. Conclusions

This study aims to investigate the spray construction process of non-water reacting polymer materials. Combined with the spraying model test, the factors affecting the spraying effect of the polymer damping layer were explored, and the spraying construction technology of the polymer damping layer was proposed. Based on this study, a suitable range of the spraying process parameters was finally obtained to guide the construction, and a demonstration was carried out on-site to verify the feasibility of the spraying technology. The main conclusions are as follows:

1. Based on the influence of environmental factors on the polymer damping spraying layer and the analysis of construction site conditions, it is found that heat affected the overall spraying effect, and the main ways of heat loss included heat exchange due to low ambient temperature, heat carried away by airflow (wind) and heat absorbed by the sprayed base wall. The above factors should be comprehensively considered in spray design and construction to minimize heat loss and ensure the spraying quality of the polymer damping layer.
2. At significantly lower/higher initial material temperature or higher relative humidity, the uniformity of the damping layer was affected, and thus the adhesive strength between the polymer damping layer and the base was reduced.
3. It is necessary to maintain the same proportion of Components A/B of the polymer materials and reasonably control the spray gun pressure and spray gun motion parameters (i.e., distance, speed and angle), in order to achieve a more uniform polymer damping layer.

It provides theoretical and technical guidance for the design and construction of the damping layer of the lining structure of mountain tunnels in strong earthquake areas. The following aspects will be considered in future research:

1. Improve polymer materials to enhance the shock absorption performance of materials.
2. Further develop automatic spraying equipment to realize automatic spraying construction with the dynamic adjustment of spray parameters, based on the obtained parameter table.

## Figures and Tables

**Table 1:** Scheme of spraying test of the polymer and parameter selection.

Influence Factors	Test Parameter Selection							
Material ratio	<1:1			1:1			>1:1	
Material temperature, °C	25		35	45		55	65	
Environment and tunnel base temperature, °C	10				25			
Humidity of tunnel base	6% (<8%)			12% (>8%)			12% (>8%) + Polyurethane primer	
Wind speed, m/s	3				7			
Spraying pressure, MPa	4	6	8	10	12	14	16	18
Spray gun speed, cm/s	25 50			75 100			125	150
Spraying distance, cm	40 60 80 100 120 140 160							
Spraying angle, °	30° 60° 90°							

**Table 2:** Effects of initial material temperatures on spraying performance.

$T_{poly}\;({}^{\circ}C)$	Tensile Adhesive Strength (kPa)	Damage Location	Sagging Situation	Splash Situation	Debonding Time (s)
25	165	Polymer	Slight	Serious	6
35	401	Polymer	None	None	5
45	418	Polymer	None	None	3
55	300	Polymer	None	None	2
65	195	Base interface	Severe exotherm	None	2

**Table 3:** Relationship between pressure value of spraying and spraying effect.

$\sigma_{spray}\;(\mathrm{MPa})$	Diffusion Radius (cm)	Atomization Effect	Sagging Situation	Splash Situation
4	6	Very bad	Serious	None
6	24	Very bad	Slight	None
8	28	Poor	Slight	None
10	32	Good	None	None
12	35	Good	None	None
14	40	Better	None	Slight
16	42	Better	None	Serious
18	45	Better	None	Serious

**Table 4:** Spray coating properties at different $v_{gun}$.

$v_{gun}\;(\mathrm{cm}/s)$	Spray Coating Situation	Single Coating Thickness (cm)	Apparent Density (kg/m^3^)
25	More uniform	1.9–2.1	512
50	More uniform	1.1–1.3	498
75	Uniform	0.7–1.0	502
100	Uniform	0.6–0.8	494
125	Uniform	0.4–0.5	522
150	Nonuniform	0.2–0.3	510

**Table 5:** Summary of spraying results at different spray gun distances.

$L_{spray}\;(\mathrm{cm})$	Diffusion Radius (cm)	Sagging Situation	Splash of Materials	Spray Coating Situation
40	35	Slight	Serious	Uneven
60	38	Slight	Serious	Uneven
80	40	Slight	Slight	Uneven
100	41	None	Slight	More uniform
120	36	None	None	Uniform
140	28	None	None	Uneven
160	26	None	None	Uneven

**Table 6:** Recommended range of spraying process parameters.

Parameter	Value (Range)
$w_{base}$ (%)	<8
$v_{wind}$ (m/s)	<5
$T_{en}$ (℃)	10–35
$T_{poly}$ (℃)	35–45
λ	1:1
$\sigma_{spray}$ (MPa)	10–12
$v_{gun}$ (cm/s)	Varying
$L_{spray}$ (cm)	100–120
$\theta_{spray}$ (°)	90

**Table 7:** Apparent quality in the spraying process and physical properties of the spray after completion.

Indicator	During Spray	After Spray
Surface crusting	None	-
Bubble or hanging flow	None	-
Debonding time (s)	≤30	-
Strength development cycle (min)	≤20	-
Density (kg/m^3^)	-	120
Thermal conductivity at 23 ± 2 °C (W/(m∙k))	-	≤0.036
Tensile adhesive strength to cement mortar (MPa)	-	≥0.10 (the damage shall not be located at the bonding interface)
Permeability	-	Impermeable

## Data Availability

All data generated or analyzed are included in this manuscript.
